# Can Red Yeast (*Sporidiobolus pararoseus*) Be Used as a Novel Feed Additive for Mycotoxin Binders in Broiler Chickens?

**DOI:** 10.3390/toxins14100678

**Published:** 2022-09-29

**Authors:** Orranee Srinual, Tossapol Moonmanee, Chompunut Lumsangkul, Hien Van Doan, Montri Punyatong, Mongkol Yachai, Thanongsak Chaiyaso, Kittima Kongtong, Wanaporn Tapingkae

**Affiliations:** 1 Department of Animal and Aquatic Sciences, Faculty of Agriculture, Chiang Mai University, Chiang Mai 50200, Thailand; 2 Innovative Agriculture Research Center, Faculty of Agriculture, Chiang Mai University, Chiang Mai 50200, Thailand; 3 Faculty of Animal Science and Technology, Maejo University, Chiang Mai 50290, Thailand; 4 Division of Biotechnology, Faculty of Agro-Industry, Chiang Mai University, Chiang Mai 50100, Thailand; 5 National Center for Genetic Engineering and Biotechnology (BIOTEC), Pathum Thani 12120, Thailand

**Keywords:** red yeast, *Sporidiobolus pararoseus*, mycotoxin binder, broilers, novel feed additive

## Abstract

Mycotoxin-contaminated feeds may negatively affect broiler chickens’ health; hence, a sustainable approach to achieve mycotoxin elimination is necessary. This study aimed to evaluate the efficacy of red yeast (*Sporidiobolus pararoseus*; RY) as a novel mycotoxin binder in broilers. A total of 1440 one-week-old male broiler chicks were randomly assigned to 12 treatments in a 3 × 4 factorial design. The dietary treatments included three levels of mycotoxin-contaminated diets (0 µg kg^−1^ (0% of mycotoxin; MT), 50 µg kg^−1^ (50% MT), and 100 µg kg^−1^ (100% MT)) and four levels of mycotoxin binders (0.0 and 0.5 g kg^−1^ commercial binder, and 0.5 and 1.0 g kg^−1^ RY). Experimental diets were contaminated with aflatoxin B1, zearalenone, ochratoxin A, T-2 toxin, and deoxynivalenol in the basal diet. Furthermore, the parameters including feed intake, body weight, and mortality rate were recorded on a weekly basis. After feeding for 28 days, blood and organ samples were collected randomly to determine the blood biochemistry, relative organ weights, and gut health. The results indicated that mycotoxin-contaminated diets reduced the average daily weight gain (ADG), villus height (VH), and villus height per the crypt depth ratio (VH:CD) of the intestine, as well as the population of *Lactobacillus* sp. and *Bifidobacterium* sp. in the cecal (*p* < 0.05), whereas they increased the mycotoxins concentration in the blood samples and the apoptosis cells (TUNEL positive) in the liver tissue (*p* < 0.01) of broiler chicken. In contrast, RY-supplemented diets had better ADG values and lower chicken mortality rates (*p* < 0.05). Moreover, these combinations positively impacted the relative organ weights, blood parameters, bacteria population, intestinal morphology, and pathological changes in the hepatocytes (*p* < 0.05). In conclusion, RY supplementation effectively alleviated the toxicity that is induced by AFB1 and OTA, mainly, and could potentially be applied as a novel feed additive in the broiler industry.

## 1. Introduction

The broiler industry is one of the most important sectors for animal protein production, with there being a global production of approximately 137 million tons in 2020 [1]. The industry contributes to produce sufficient quantities of high-quality animal protein to meet the rapidly growing consumption needs of the human population [1,2]. Due to the high demand from local and international markets, the broiler industry has expanded and intensified globally over the past few decades. However, the presence of antinutritional agents and toxins in feeds has caused a slew of problems for the broiler industry, including slower growth rates, poor feed quality, and higher mortality rates [1]. Fungus spoilage is the most common cause of poultry feed contamination [1]. Fungus thrives in environments where the harvesting methods are poor, the ambient temperatures are high, and the cereals have not been properly dried in the field [3]. As a result, secondary fungal metabolites that are linked to mycotoxin production are an increasing trend [1,4]. The contamination of farm animal feeds with mycotoxins results in substantial economic losses [5,6]. Worldwide, mycotoxins have caused severe effects in about a quarter of all crops, and lead to damages that cost the US agricultural economy USD 1.4 billion annually [5].

Mycotoxins are a convenient generic term for the toxic substances that are produced by fungi during their development [7,8]. They can occur during crop growth, the post-harvest storage period, or in compound feed storage [9]. Mycotoxins are fungal metabolites that can contaminate a wide range of food and feed products, thereby potentially harming poultry and livestock [10]. Mycotoxin contaminations are a global concern in agricultural products and feedstuffs [8,11,12]. Among the major agriculture-related mycotoxins, aflatoxin B1 (AFB1), zearalenone (ZEA), deoxynivalenol (DON), fumonisins (FB), T-2 toxin (T-2), and ochratoxin (OTA) have been found to be the most prevalent [13]. In general, the signs of toxicity from mycotoxins in animals include them exhibiting a decreased level of production, weight, feed conversion, and feed intake, increased levels of bloody diarrhea, severe dermatitis, hemorrhages, and an increased death rate [5,12]. Furthermore, this causes reproductive problems and developmental toxicity in poultry and histopathological changes in the kidneys and liver [6,14,15]. Moreover, mycotoxins that are produced by the *Fusarium* spp. fungus is known to induce apoptosis in mammalian cells. Trichothecene mycotoxin causes acute and chronic toxicity and induces apoptosis in the immune system and liver tissues, especially AFB1 and OTA [8,16]. In addition, OTA exhibits a cytotoxic effect on the mucosa-associated lymphoid tissue and intestinal epithelium, which alters the intestinal barrier and increases the animal’s susceptibility to various related diseases that cause to a reduced rate of nutrient absorption [17]. Furthermore, they have no distinct odor and do not change the organoleptic properties of the foods that they are in [13].

Research on mycotoxin removal from contaminated foods and foodstuffs has recently focused on degrading, destroying, inactivating, or removing the mycotoxins by using physical, chemical, nutritional, or biological methods [18]. The most effective methods for minimizing mycotoxin harm in animal husbandry use particular materials that adsorb mycotoxins, thus limiting their bioavailability in the body [19]. These substances, including inorganic adsorbents (e.g., clays, bentonites, aluminosilicates), natural phytochemicals from plants [20,21], and organic compounds (e.g., yeast or bacterial cell walls), were very effective against aflatoxigenic, ochratoxigenic fungi, and AF and OTA accumulation in maize-based mediums and they can efficiently bind with AFB1 [22]. Clays are porous, inorganic materials that contain silicate tetrahedron rings. Mycotoxins can be adsorbed and trapped by electric elementary charges in this porous structure. The majority of them are known to be effective AFB1 binders more than 90%. However, clays are very limited when they are working against the fusariotoxins such as ZEN, T-2, DON, and FB [8]. However, the addition of such adsorbents to mycotoxin-contaminated diets could decrease their bioavailability in the digestive tract, as well as their negative effects on animals. 

The yeast (*Saccharomyces cerevisiae*) cell wall has been widely used as a mycotoxin binder in the broiler industry [23,24,25]. The yeast cell walls consist of polysaccharides (glucan, mannan) which function to absorb mycotoxins via different binding mechanisms [26,27]. Recently, Yiannikouris et al. [28] reported that the ß-D-glucan component of the yeast cell wall is directly implicated in the binding process of mycotoxins and modulates their binding strength. The glucan–mycotoxin complexes have been found to have hydrogen and van der Waals bonds, and they are stable at the pH condition of the digesta all along the digestive tract. Haque et al. [8] demonstrated that the yeast cell wall can efficiently adsorb more than 90% of the AFB1. Moreover, the yeast cell wall showed that it could cause a potential deactivation of ZEA and OTA in animal feeds [29]. Therefore, organic binders are efficient against a larger range of mycotoxins than inorganic binders are; they are biodegradable and do not accumulate in the environment after being excreted by animals [29].

Red yeast (*Sporidiobolus pararoseus* KM281507) is a novel yeast that has been shown to naturally produce the ß-carotene pigment [26,30]. Carotenoids defend the cells and other body components from free radical attacks by acting as chain-breaking anti-oxidants [31]. The biological damage that is caused by oxidative stress which is caused by free radicals is harmful at the cellular level, and it has been linked to health issues that affect animal productivity [32,33]. In addition to their high nutritional value, the cell wall’s component of red yeast can function as mycotoxin binders [28,34] and improve the productive performance of chickens [26,35,36]. Tapingkae et al. [37] demonstrated that the new strain of red yeast that is capable of binding ZEA, AFB1, OTA, and trichothecenes has been commercially used to adsorb mycotoxin in animal and poultry diets. The red yeast cell wall was able to bind ZEA, AFB1, OTA, DON, and T-2 by simulating a gastrointestinal model of poultry, and the result of these studies is that they were 99, 93, 79, 72, and 59% effective, respectively. However, no available data regarding the use of red yeast as a mycotoxin binder in broilers have been reported. Therefore, the current trial was designed to investigate the effects of red yeast as a mycotoxin adsorbent on growth performance, blood parameters, relative organ weight, the microbial population in cecal, the small intestinal morphology, and liver cell apoptosis in broilers when they received a dietary mycotoxin contamination.

## 2. Results

### 2.1. Growth Performance

Data for the combined grower (8 to 21 day)–finisher period (21 to 35 day) and the overall period (8 to 35 day) are presented in Table 1. During the grower phase (d 8 to 21), the body weight, ADG, FCR, and mortality rates were unaffected by the dietary treatment (*p* > 0.05), while the ADFI was reduced in both of the mycotoxin-contaminated groups when compared with that of the control group (*p* < 0.05). At 21–35 day, the mortality rates were increased among the birds that were fed the mycotoxins diets (*p* < 0.001). In contrast, supplementing a binder with the contaminated diet decreased the mortality rates considerably (*p* < 0.001).

For 28 days (8 to 35 day) of the toxin administration period, an interaction was observed between the final weight, the ADG, and the mortality rate, depending on what the toxin level was and the inclusion or exclusion of adsorbents. During this period, reductions (*p* < 0.05) in the final weight and the ADG were observed in the mycotoxin administration groups when they were compared with the control group. Nonetheless, the addition of the red yeast (RY) compound resulted in higher final weight and ADG, in contrast to the mycotoxin groups. In terms of the mortality rates, the mortality rate was significantly increased in the 100% toxin inclusion group (*p* < 0.001) when it was compared with those of the other groups, whereas the adsorbent-supplemented diet significantly reduced (*p* < 0.001) the mortality rates as compared with those that resulted from using all other toxin levels.

### 2.2. Blood Parameters

#### 2.2.1. Blood Chemistry

During the toxin administration period, no interactions (*p* > 0.05) between the mycotoxin levels and the adsorbent groups occurred in terms of alanine aminotransferase, albumin, alkaline phosphatase, creatinine, total protein, blood urea nitrogen, and superoxide dismutase in the serum (Table 2). However, it was found that the main effect of mycotoxins was significant (*p* < 0.05) in the increase that it had on alanine aminotransferase (ALT) in the serum. 

#### 2.2.2. Mycotoxin Determination in Blood Samples

Concentrations of AFB1, ZEN, OTA, T-2, and DON were detected in the plasma samples of the mycotoxin-contaminated group in relation to the increasing mycotoxin concentrations that were in the diets, and in most cases, there were significantly (*p* < 0.01) decreased concentrations in the adsorbent groups (Table 3). The mycotoxin concentration also increases in the broilers that were fed only the mycotoxins that were at concentrations of 0.45 and 1.26 ng AFB1 mL^−1^ (R^2^ = 0.981), whereas ZEN was detected only in the samples of the mycotoxin group with increasing concentrations of them of 1.13 and 2.74 ng mL^−1^ (R^2^ = 0.979). In addition, the individual ratios of the OTA in the plasma samples were approximately 4.32 ng mL^−1^ in the ones with the highest level of mycotoxin contamination (R^2^ = 0.965). Moreover, the highest concentrations of trichothecenes were found in the plasma samples, and the concentration was characterized by a high level of trichothecene toxins at 1.04 ng T-2 mL^−1^ and 1.08 ng DON mL^−1^ (R^2^ = 0.990 and 0.983, respectively). 

### 2.3. Relative Organ Weight

The effects of the mycotoxins and the mycotoxin plus adsorbent-supplemented diets on the relative organ weight are presented in Table 4. The relative weights of the kidneys, livers, and pancreases were at their highest in the 100% mycotoxin diet group (*p* < 0.05). Intriguingly, a significant decrease in the kidney, liver, and pancreas weights was only found in the diets that included the adsorbent (*p* < 0.01).

### 2.4. Apoptotic Cells in Liver Tissue

**Figure 1:**
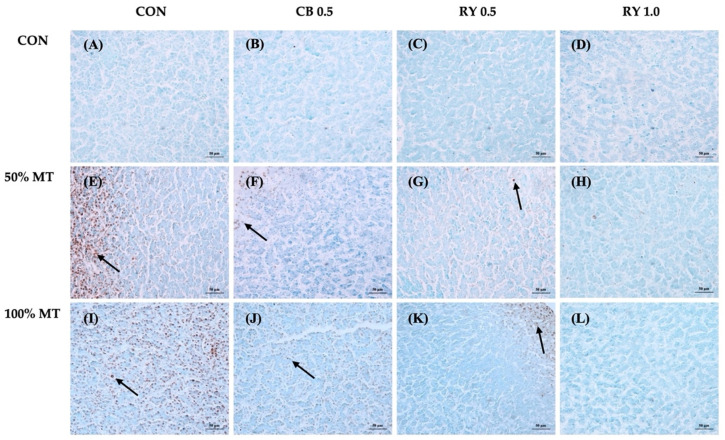
The apoptosis cells in the liver tissue were detected using TUNEL staining. MT: mycotoxin-contaminated diets; CB: commercial mycotoxin binder supplementation; RY: red yeast supplementation. (**A**) Control diet (CON); (**B**) CB at 0.5 g kg^−1^; (**C**) RY at 0.5 g kg^−1^; (**D**) RY at 1.0 g kg^−1^; (**E**) MT 50% (50 ppb); (**F**) MT 50% + CB at 0.5 g kg^−1^; (**G**) MT 50% + RY at 0.5 g kg^−1^; (**H**) MT 50% + RY at 1.0 g kg^−1^; (**I**) MT 100% (100 ppb); (**J**) MT 100% + CB at 0.5 g kg^−1^; (**K**) MT 100% + RY at 0.5 g kg^−1^; (**L**) MT 100% + RY at 1.0 g kg^−1^. Many TUNEL-positive cells (black arrow) with brown reaction products in the nuclei were present at the liver cells. Magnification was 10 × the objective lens. Scale bars represent 50 µm.

In the livers of the broiler that were fed the mycotoxin-contaminated diet, positive staining was observed in hepatocytes areas of the livers (Figure 1). Because the mycotoxin-contaminated diet group had pathology lesions in their livers, we examined the liver for apoptosis by the TUNEL-positive cells. Specifically, the libers of the broilers that were fed the mycotoxin-contaminated diet showed had a widespread positive staining of the hepatocytes area (dark brown), and this interaction showed that red yeast might decrease mycotoxin cytotoxicity.

**Figure 2:**
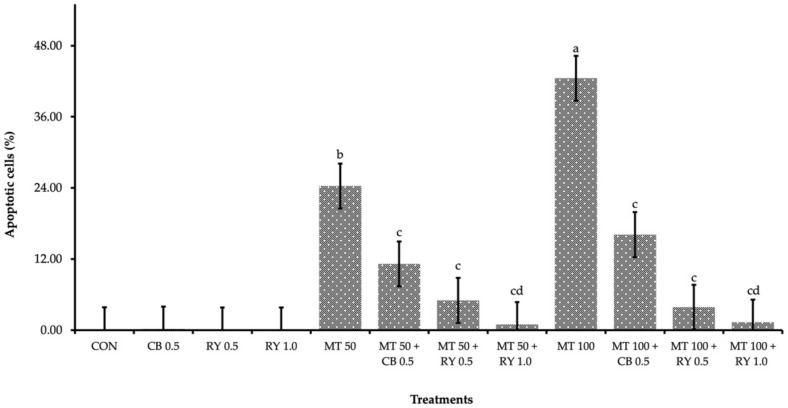
The percentage of apoptotic cells (TUNEL-positive) was counted in 10 randomly selected high-power fields from 6 livers per group. On each bar, significant differences at *p* ≤ 0.05 levels are indicated by the different lowercase letters (a, b, c, and d), while insignificant differences at *p* > 0.05 levels are indicated by the same lowercase letters.

Moreover, these results suggested that apoptosis that was induced by mycotoxin poisoning may have contributed to the increasing in total percentage of apoptosis cells that were observed in the mycotoxin-contaminated group (Figure 2). In contrast, in the diets that included the adsorbent, the percentage of positive nuclei in the mycotoxins in the livers was significantly more decreased than that of only mycotoxin-contaminated diet. Especially, the experiment with group for which 1.0 g kg^−1^ of red yeast was supplemented resulted in the lowest percentage of positive nuclei (*p* < 0.001).

### 2.5. Cecal Microbial Populations

Table 5 shows the populations of cecal microflora in the broiler chickens. The highest level of mycotoxins that was in the diet resulted in the there being the largest population of *Escherichia coli* (*p* < 0.001), whereas *Lactobacillus* sp. and *Bifidobacterium* sp. populations were the least common types (*p* < 0.01). The addition of adsorbent compounds to the mycotoxin diet reduced the population of the pathogens to a level that was comparable with the control diet (*p* < 0.001). Furthermore, *Lactobacillus* sp. and *Bifidobacterium* sp. populations tended to increase in the chicks that were fed diets with 0.5 and 1.0 g kg^−1^ RY (*p* < 0.01) as compared to the feeding of chicks with individual mycotoxin diets.

### 2.6. Intestinal Morphology

#### 2.6.1. Intestinal Morphology in the Duodenum

**Figure 3:**
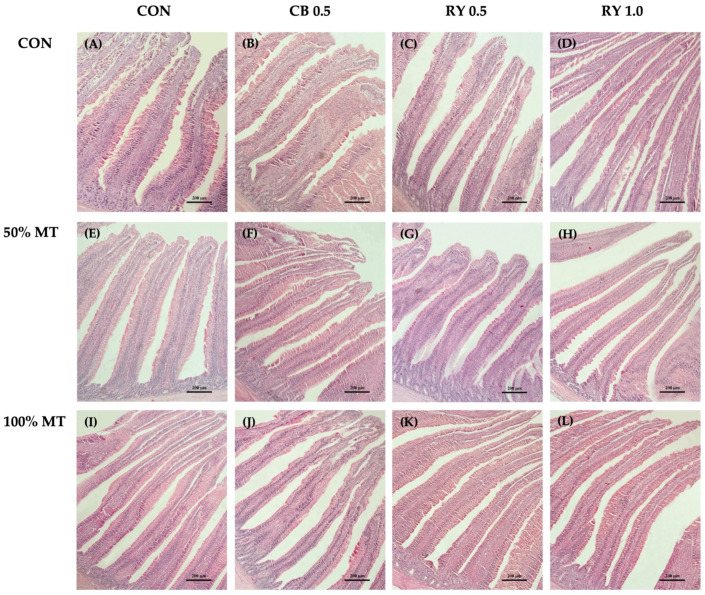
Histological representations of the H and E-stained duodenal sections of broiler chickens. MT: mycotoxin-contaminated diets; CB: commercial mycotoxin binder supplementation; RY: red yeast supplementation. (**A**) Control diet (CON); (**B**) CB at 0.5 g kg^−1^; (**C**) RY at 0.5 g kg^−1^; (**D**) RY at 1.0 g kg^−1^; (**E**) MT 50% (50 ppb); (**F**) MT 50% + CB at 0.5 g kg^−1^; (**G**) MT 50% + RY at 0.5 g kg^−1^; (**H**) MT 50% + RY at 1.0 g kg^−1^; (**I**) MT 100% (100 ppb); (**J**) MT 100% + CB at 0.5 g kg^−1^; (**K**) MT 100% + RY at 0.5 g kg^−1^; (**L**) MT 100% + RY at 1.0 g kg^−1^. Magnification was 10× the objective lens. Scale bars represent 200 µm.

In the duodenum morphology (Table 6; Figure 3), the villi were consistently longer in a 1.0 g kg^−1^ RY-supplemented diet when they was compared to those of other groups (*p* < 0.001). In contrast, the broilers that were fed mycotoxin-contaminated diets at 50% and 100% concentrations showed a significant decrease (*p* < 0.05) in their villus height (VH) when they were compared with those of the birds that were fed an RY-supplemented diet. The villus width (VW) was smaller in the broilers that were fed either a 50% mycotoxin-contaminated diet or the control diet (*p* < 0.05). However, the duodenum’s VW was widest in the contaminated diets when a 50% concentration of mycotoxins was added to the commercial mycotoxin binder with 0.5 g kg^−1^ RY as compared with the control group (*p* < 0.001). The broilers that were fed the mycotoxin-contaminated diet at a 100% concentration throughout the feeding trial had deeper crypts (*p* < 0.001). In contrast, the broilers that received only 1.0 g kg^−1^ RY in their diet had shallower crypts in the duodenum, yet the differences were not statistically significant when they were compared with the control group (*p* < 0.01). The muscularis mucosae thickness (MMT) of the duodenum was significantly higher in the group that was fed the 50% mycotoxin-contaminated diet when it was compared with that of the control group (*p* < 0.001).

Moreover, the results indicated that the duodenal villus height per crypt depth ratio (VH:CD) was significantly higher in the 1.0 g kg^−1^ RY-supplemented group when it was compared to that of the control group (*p* < 0.01). However, the VH:CD ratio was significantly lower (*p* < 0.01) in the group that was fed the 100% mycotoxin-contaminated diet when it was compared to that of the control group. There were statistically higher absorptive surface area (VSA) values which were noticed in the broilers that were given mycotoxin plus the adsorbent in a 50% mycotoxin-contaminated diet (*p* < 0.05) in comparison to those of the control group. In contrast, the broilers that were fed the 100% mycotoxin-contaminated diet had the lowest (*p* < 0.05) VH:CD ratio and VSA values.

#### 2.6.2. Intestinal Morphology in the Jejunum

**Figure 4:**
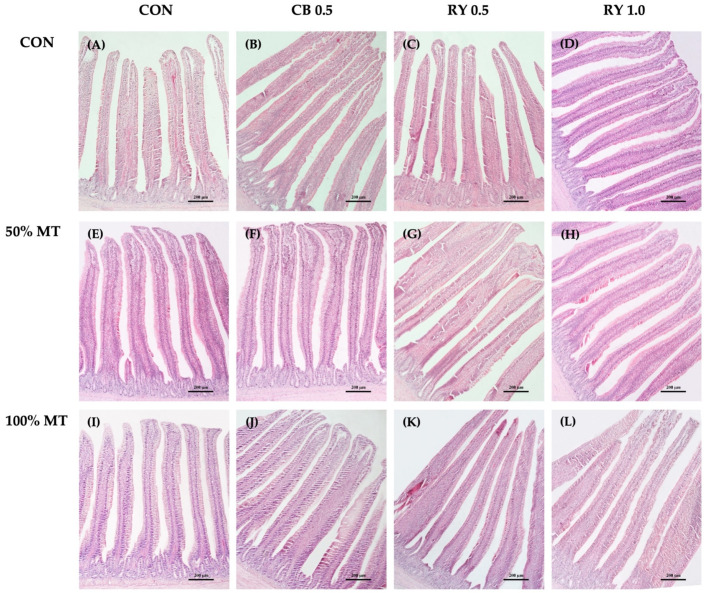
Histological representations of the H and E-stained jejunal sections of broiler chickens. MT: mycotoxin-contaminated diets; CB: commercial mycotoxin binder supplementation; RY: red yeast supplementation. (**A**) Control diet (CON); (**B**) CB at 0.5 g kg^−1^; (**C**) RY at 0.5 g kg^−1^; (**D**) RY at 1.0 g kg^−1^; (**E**) MT 50% (50 ppb); (**F**) MT 50% + CB at 0.5 g kg^−1^; (**G**) MT 50% + RY at 0.5 g kg^−1^; (**H**) MT 50% + RY at 1.0 g kg^−1^; (**I**) MT 100% (100 ppb); (**J**) MT 100% + CB at 0.5 g kg^−1^; (**K**) MT 100% + RY at 0.5 g kg^−1^; (**L**) MT 100% + RY at 1.0 g kg^−1^. Magnification was 10× the objective lens. Scale bars represent 200 µm.

There was no significant effect on the mycotoxins and mycotoxin binder levels on the VW and VSA (Table 7; Figure 4). However, that broilers that were fed commercial mycotoxin binders and 1.0 g kg^−1^ RY had a significantly higher VH than the control group did (*p* < 0.001). The VH in the jejunum of the broilers that were fed the 50% and 100% mycotoxin-contaminated diets were significantly (*p* < 0.001) lower than those of the groups that were administered the mycotoxin binder. Moreover, the broilers that were fed a 100% mycotoxin-contaminated diet throughout the feeding trial had the deepest crypts (*p* < 0.01) when they were compared with those of the control group. In contrast, the broilers that received a combination of a 50% mycotoxin-contaminated diet with a 0.5 g kg^−1^ commercial mycotoxin binder and RY had shallower crypts in the jejunum when they were compared with those of other groups (*p* < 0.01). The MMT of the jejunum was significantly higher in the 100% mycotoxin-contaminated groups than it was in the control group (*p* < 0.001). The RY-supplemented diet appeared to decrease the MMT in comparison to the effect of the mycotoxin-contaminated diets (*p* < 0.001). Moreover, the histological results showed that the addition of the mycotoxin binder significantly increased (*p* < 0.05) the VH:CD ratio in the jejunum when it was compared with that of the mycotoxin-contaminated groups.

#### 2.6.3. Intestinal Morphology in the Ileum

**Figure 5:**
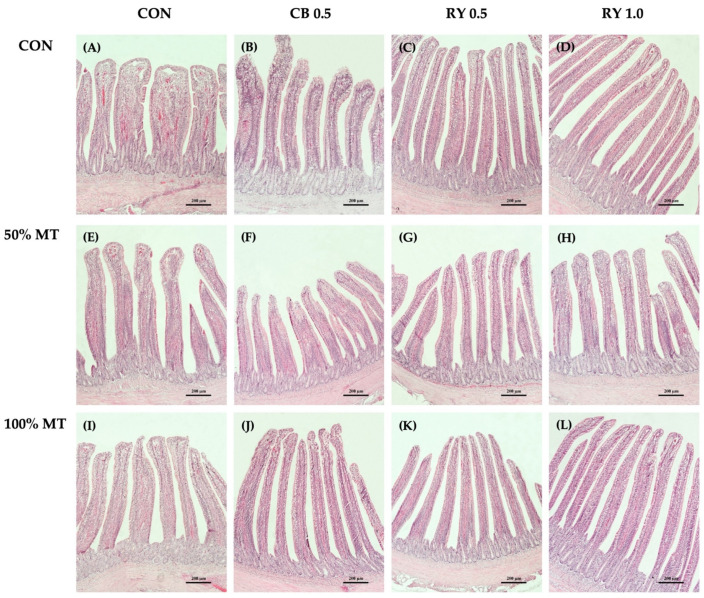
Histological representations of the H and E-stained ileal sections of broiler chickens. MT: mycotoxin-contaminated diets; CB: commercial mycotoxin binder supplementation; RY: red yeast supplementation. (**A**) Control diet (CON); (**B**) CB at 0.5 g kg^−1^; (**C**) RY at 0.5 g kg^−1^; (**D**) RY at 1.0 g kg^−1^; (**E**) MT 50% (50 ppb); (**F**) MT 50% + CB at 0.5 g kg^−1^; (**G**) MT 50% + RY at 0.5 g kg^−1^; (**H**) MT 50% + RY at 1.0 g kg^−1^; (**I**) MT 100% (100 ppb); (**J**) MT 100% + CB at 0.5 g kg^−1^; (**K**) MT 100% + RY at 0.5 g kg^−1^; (**L**) MT 100% + RY at 1.0 g kg^−1^. Magnification was 10× the objective lens. Scale bars represent 200 µm.

There was a statistical response in the ileal morphology (Table 8; Figure 5) that was due to the treatments (*p* < 0.001). The broilers that were fed a 0.5 g kg^−1^ RY diet had the highest VH in comparison with the control group, whereas the broilers that received the mycotoxin-contaminated diets and the commercial mycotoxin binders in the 50% contaminated diets had shorter VH (*p* < 0.001). Additionally, the broilers that were fed the 100% mycotoxin-contaminated diets had narrow villi in the ileum region, deeper crypts, and a higher MMT (*p* < 0.05) when they were compared with those of the control group. However, the MMT in the ileum was significantly decreased in the broilers that received the mycotoxins in combination with the RY-supplemented diet (*p* < 0.001) when it was compared with that of the control group. Moreover, the broilers that received the mycotoxin (100%) diet for 28 days had the lowest VH:CD ratio in the ileum when it was compared with those from the broilers that were fed the mycotoxin with the mycotoxin plus adsorbent diets (*p* < 0.05). In contrast, the RY-supplemented diets appeared to increase the VH:CD ratio when these results were compared to the control group, while their bases were significantly higher (*p* < 0.001) in comparison to the mycotoxin-contaminated diets that were applied in combination with the 0.5 g kg^−1^ RY diet.

## 3. Discussion

Mycotoxins cause huge economic losses and diseases in livestock farming [6]. Among these farmed animals, poultry are the most vulnerable and sensitive to these mycotoxins. Furthermore, the consumption of a mycotoxin-contaminated diet has been reported to reduce the growth performance of them and lead to enhance the pathology characteristics in broilers [1,6,8]. Therefore, the removal of mycotoxins using sustainable and environmentally friendly toxin binders in the poultry industry is an urgent issue.

### 3.1. Growth Performance

In the present study, a reduction in the average daily weight and an increased mortality rate were noticed in the broilers that were fed mycotoxin-contaminated diets without toxin binders. Similar results were found in previous studies in chickens that were fed mycotoxin diets [1,25,38,39,40]. This may be attributed to the synergistic effects of different mycotoxins [38]. In general, livestock animals are exposed to two or more types of mycotoxins simultaneously, rather than a single mycotoxin [6]. Therefore, the ingestion of mycotoxins in contaminated feed could cause various health problems in animals, including a reduction in their immune function, organ damage, as well as problems in the nervous and reproductive systems, all of which may contribute to poor growth efficiency [41]. Nevertheless, the addition of a toxin binder (red yeast) to the mycotoxin-contaminated diets in our study resulted in a higher ADG and a lower mortality rate when they were compared with those that resulted from the contaminated diets without toxin binders. The results coincided with the previous findings by Arif et al. [1] and Weaver et al. [25] who stated that the addition of a yeast cell wall to a natural mycotoxin-contaminated diet significantly reduced the negative effects of the mycotoxins on body weight gain, feed intake, and the feed conversion ratio in broilers. This improvement may be attributable to the beneficial effects of the yeast cell wall which has been proven to be a biodegradable adsorbent with prebiotic and antioxidant properties [1,42]. It has been acknowledged that the ß-D-glucans from yeast cells could act as efficacy mycotoxin binders [24]. Similar effects have previously been reported by Tapingkae et al. [37] who reported that red yeast exhibited the capacity to adsorb mycotoxins including aflatoxin B1 (AFB1), ZEA, OTA, T-2, and DON. Moreover, the red yeast–mycotoxin complex is stable in the broilers’ gastrointestinal tract [37], thereby resulting in a reduced impact of mycotoxins on the broilers’ growth performance.

### 3.2. Blood Parameters

Regarding the hematological parameters, the present study indicated that diet contamination and binder addition did not affect the serum biochemical parameters in the broilers. The result was in accordance with the previous study by Dänicke et al. [43] who found that there were no effects on these parameters in a study where they fed a natural mycotoxin diet to broilers for 35 days. However, the failure to obtain any significant effect of the contaminated diets on the blood chemistry in the current study may be due to the short duration of the exposure of the birds to relatively low levels of dietary mycotoxin content [40]. In contrast, the main effect of the mycotoxins was the significant effect that they had on alanine aminotransferase (ALT) in the broilers that were serum-fed the contaminated diets. The presence of these enzymes at higher concentrations is a biomarker of liver dysfunction and is thought to indicate damage to the membrane integrity of the liver cells [25,44]. Several studies have demonstrated that mycotoxin exposure results in liver function damage with there being significantly increased ALT levels in the serum [25,44,45]. Hepatocytes with an altered lipid metabolism and signaling pathways may be responsible for these consequences [44], according to the results of a study of the liver’s relative weight. In the same way, the present study was carried out to examine the dependent effects of mycotoxins and to assess the relationship between the broilers’ exposure to mycotoxins and the adsorbent concentrations of AFB1, ZEN, OTA, T-2, and DON in their blood samples. The quantified mycotoxin concentrations in the serum are similar to the findings of Brezina et al. [46] and Lauwer et al. [47] who animals fed a 50–200 ug kg^−1^ diet of mycotoxin which corresponded to a concentration of 0.5–15.0 ng mL^−1^ in the blood samples, which could be due to the lack of an intestinal de-epoxidation ability in some animals [46]. Additionally, Brezina et al. [46] reported that mycotoxins that have been consumed are almost completely absorbed in the upper gastrointestinal tract before the microbial de-epoxidation of them, which explains the lack of or low concentrations of mycotoxins in these blood samples.

### 3.3. Relative Organ Weight and Apoptotic Cells in Liver Tissue

Our findings showed that the application of mycotoxin-contaminated diets without the toxin binders resulted in a significant increase in the relative weights of the livers, kidneys, and pancreases. These findings were in accordance with the previous studies by Arif et al. [1], Mazur-Kuśnirek et al. [48], and Saminathan et al. [45] who indicated that there was a significant increase in the relative weight of livers, kidneys, and pancreases. The enlargement of the liver and kidneys is commonly seen in mycotoxin exposure [49]. The increase in the relative weight of the livers as it is induced by mycotoxins is attributed to an accumulation of lipids in the liver, which produces the characteristic, enlarged, and friable fatty livers [49]. The increase in liver weight in the broilers can be indicative of the initial phase of an inflammatory response because the liver is the site for the manufacture of acute phase proteins that cause lipid peroxidation in tissues [50]. Therefore, we confirmed that mycotoxins demonstrate an inhibitory effect on the growth of hepatocytes cells, thereby causing damage and promoting apoptosis in hepatocytes cells. The molecular mechanisms that are involved were explored, and the OTA and AFB1 had synergistic cytotoxic effects by inducing apoptosis in the liver cells [16,17]. Wang et al. [17] reported that the OTA could increase cell apoptosis in cultured hepatocytes (PHHs) and Kupffer cells (HKCs), and it could possibly activate the JNK signal pathway mechanism and affect the expression of caspase-3, Bax, and Bcl-2 in cell death. Moreover, in the toxic cells with an innately high level of intracellular reactive oxygen species (ROS) [51], the ROS has both pro- and anti-tumorigenic roles in cancer cells. At low concentrations, an increase in cellular ROS can trigger pro-tumorigenic signaling, thereby enhancing cell proliferation and increasing apoptosis cell [51]. In the current study, the supplementation of red yeast showed significant protective effects with respect to organ damage, as indicated by the inhibition of liver and kidney enlargement. These findings are in line with a previous study [52], which showed that the supplementation of the inactive yeast cell wall can help to mitigate the combined effects of AFB1, DON, and OTA on the health status of laying hens. In contrast, the supplementation of the yeast cell wall could only prevent improvements in the liver and spleen indices. However, the mode of action of the yeast cell walls in decreasing the liver weight is not clear. This could be related to both the yeast cell wall’s ability to absorb mycotoxins and the carotenoid component of red yeast to lessen the toxicity levels. Carotenoids have the potential to behave as strongly pro-oxidant agents and reduce the apoptosis which is mediated by ROS. Carotenoids perform a variety of dynamic activities that can improve the oxidative stress in healthy cells while worsening the oxidative stress in cancer cells, and thereby resulting in the reduction of apoptosis cells in liver tissue [51]. These findings are in line with a previous study by Kittichaiworakul et al. [53], wherein, red yeast was reported to have an antigenotoxic potential on AFB1-induced mutagenesis in rat livers and *Salmonella Typhimurium*. The inhibitory mechanism of red yeast might be involved in the modulation of xenobiotic metabolizing enzymes in AFB1 metabolism. In addition, carotenoids in red yeast were considered to be potential cancer chemo-preventive agents in red yeast. Therefore, the supplementation of red yeast demonstrated that it protects the broilers against the negative effects of organ damage, as indicated by the inhibition of liver and kidney enlargement. 

### 3.4. Cecal Microbial Populations

The toxicity of mycotoxins begins in the gut. Moreover, it is also the location of mycotoxin absorption, which results in the systemic exposure to these toxins. There are several reasons to investigate mycotoxin–gut microbiota interactions [54]. In the present experiment, a significant increase in *E. coli* and a decrease in the lactic acid bacteria count were observed in the chickens that were fed the mycotoxins. This finding is similar to previous investigations by Jahanian et al. [55] and Śliżewska et al. [56] who found that dietary mycotoxins increased the total amount of negative bacteria and reduced *Lactobacillus* sp. and *Bifidobacterium* sp. in poultry when these findings were compared to the control group. The changes that occurred in the gut microbiota population correspond to the impact of mycotoxins on the gut microbiota [54,57]. The composition of the microbiome varies depending on which part of the gut is being studied. The effects of mycotoxins on the gut microbiota are difficult to characterize, and the findings may differ depending on the study’s experimental design [57]. Because of their fungal origin, the antimicrobial properties of mycotoxins were hypothesized as soon as these compounds were purified. Thus, mycotoxins were screened for their activities against gram-positive bacteria, such as *Lactobacillus* sp. and *Bifidobacterium* sp. and induced Gram-negative bacteria such as *E. coli* [58]. Because the toxicity of mycotoxins on cells causes an increase in the mucus and gut secretions, resulting in significant environmental changes in which microorganisms grow, the population balance is disrupted, thereby leading to dysbiosis. The changes that occur in the health status that corresponds to these alterations can be responsible for bacterial translocation and increase the pathogenic population in the gut [54]. In contrast, the dietary supplementation of toxin binders resulted in decreased amounts of pathogenic bacteria in the broilers. In the cecum in particular, 1.0 g of red yeast kg^−1^ suppressed the *E. coli* population, while it increased the *Lactobacillus* sp. and *Bifidobacterium* sp. population. This result supports other researchers’ previous reports, such as Jahanian et al. [55], in which feeding the yeast cell wall components (MOS and β-glucan) to chickens that were exposed to aflatoxins decreased the ileal enumerations of *E. coli*, *Salmonella*, *Klebsiella*, and the total number of negative bacteria in the aflatoxin-challenged broiler chicks. This could be due to the cell wall of *Sporidiobolus pararoseus*, which is composed of an inner layer of insoluble ß-D-glucans and mannan-oligosaccharide (MOS) which are arranged in a network [36,37]. The cell wall components from red yeast have been reported to reduce cecum and crop pathogen populations, possibly due to the direct blockage of bacterial fimbriae, but more likely due to immune-mediated mechanisms and an increased mucosal integrity [59]. Thus, an increase in the proportions of *Lactobacilaceae* and *Bifidobacteriaceae* in the gut microbiome of mycotoxin-fed broilers, as well as an increase in the amount of Gram-positive bacteria in the broilers that were fed diets with red yeast cell walls, could indicate an induced adaptation of the microbiome and of the broiler itself to better handle mycotoxin challenges.

### 3.5. Intestinal Morphology

Moreover, mycotoxin exposure appeared to have cumulative adverse effects on the broilers’ intestinal mucosa structures. In the present study, there was a 50 and 100% decrease in the VH and VH:CD ratio, respectively, in the duodenum, jejunum, and ileum with decreasing levels of mycotoxins in the diet. Similarly, the mycotoxin contamination increased the CD, MMT, and VSA values. The result was supported by the previous reports of [60,61], in which the authors indicated that broilers which were fed DON and AFB1-contaminated diets had decreased VH and VH:CD values in the jejunum. Moreover, Applegate et al. [62] reported a linear increase in the CD values in the distal jejunum of the layers with an AFB1-contaminated diet. The villi being shortened is probably due to the impairment of cell proliferation [63]. Our data agree with previous studies in broiler chickens that showed a significant decrease in the VH in the small intestine after the ingestion of mycotoxin-contaminated diets [64]. Especially, the OTA and AFB1 have a directly toxic effect on the intestinal epithelium, which alters the intestinal barrier and decreases the morphological development of the intestinal area [17]. Additionally, the results indicated that a multi-mycotoxin intake during the starter phase is sufficient to affect the intestinal mucosa structures. The gastrointestinal tract is the site of nutrient digestion and absorption, and a fully functioning, healthy intestine is essential for fast-growing broilers to achieve maximum growth rates with superior feeding efficiency [64]. Feeding broilers mycotoxin-contaminated diets is known to reduce their VH, and it is associated with a reduction in nutrient digestion and absorption [65]. Although dietary nutrient digestibility was not directly measured in the present study, the negative effects that the mycotoxins had on the intestinal mucosa structures suggested that there would be a reduction in the rate of nutrient absorption. This hypothesis is supported by our observed reduction in feeding efficiency, which led to there being issues with growth performance. These findings further support the idea that red yeast cell walls can absorb mycotoxins, improve gastrointestinal tract health, and reduce the negative effects that are felt by broilers that are fed contaminated diets. When the broilers consumed a diet with 0.5 and 1.0 g kg^−1^ red yeast during the mycotoxin challenge, the impacts of the mycotoxins on the small intestine were reduced. At the end of the experiment, the inclusion of red yeast had a more significant impact on improving the duodenum, jejunum, and ileum structures during the mycotoxin challenge. These results may indicate that a higher product inclusion rate may have been necessary to significantly reduce the negative effects of the mycotoxins on the intestine. A similar effect of different amounts of product inclusion was observed by Weaver et al. [25], who found that broilers that were fed mycotoxin and 0.4% yeast cell wall had an increased VH. Moreover, the reduction in the duodenal and jejunal CD in animals that were fed red yeast cell walls may indicate that the additive could enhance the enterocyte survivability, thus reducing the crypt cells’ proliferative rate. On the other hand, the tendency to increase the villus width may be an adaptation strategy to increase the absorptive surface area after the damage that is caused by mycotoxins [24].

## 4. Conclusions

In summary, our study was the first to show that red yeast could minimize the deleterious effects of mycotoxins in broiler diets. Based on the present results, it can be concluded that the supplementation of a mycotoxin-contaminated diet with 0.5 and 1.0 g kg^−1^ red yeast as an adsorbent could enhance the broilers’ performance, their blood chemistry, the concentrations of mycotoxins in their blood samples, the cecal microbiota, the pathological changes in the hepatocytes, and their gut health. 

## 5. Materials and Methods

### 5.1. Preparation of the Red Yeast

The oleaginous red yeast (*Sporidiobolus pararoseus* KM281507) was prepared in the yeast malt medium (YM) medium, and cultivation conditions were reported in Chaiyaso et al. [66]. The initial pH of the medium was adjusted to 5.63 before its sterilization at 121 °C for 15 min. After cooling, this medium was transferred to cultivate the red yeast cells in the 5-L, 30-L, and 300-L bioreactors (BE Marubishi Co., Ltd., Pathum Thani, Thailand), as described by Tapingkae et al. [37]. After cultivation in a 300-L stirred tank bioreactor for 3 days using a semi-control pH strategy, the cultivated medium containing the red yeast cells was stored at 4 °C for 14 days to allow the autolysis and precipitation of red yeast cells. The red yeast cells were mixed with corn starch which was the carrier (16% *w*/*v*). Then, the mixture was subjected to a drying process at 60 °C for 24 h to obtain the red yeast powder. Therefore, red yeast powder is inactive yeast (dead yeast cells).

### 5.2. Ethical Approval and Informed Consent

All of the experiments were carried out according to the standards for the treatment and use of laboratory animals, which were approved by Maejo University, Chiang Mai, Thailand (permit number: MJUAN2560/14). All of the experiments were performed in accordance with the appropriate guidelines and regulations.

### 5.3. Animal Management

A total of 1440 eight-day-old male Cobb broiler chicks were used for the study. Chicks were fed three levels of mycotoxin (0%, 50%, and 100%) and four levels of fungal toxin adsorption (0.5 g kg^−1^ commercial mycotoxin binder and 0, 0.5, and 1.0 g kg^−1^ red yeast) for 28 days. The 3 × 4 factorial designs were used with twelve treatments and six replicates of twenty birds each. The corn-soybean meal was used as a basal diet (Table 9). The basal diets were formulated to contain adequate concentrations of all of the nutrients required for broilers according to the National Research Council recommendations in 1994 [67] and no antibiotics were added to the feed. Throughout the study, the feed and water were provided *ad libitum*. Chicks were placed on the ground during the first week of the experiment. The house temperature was regulated using an evaporative cooling system and maintained at 18–25 °C. The lighting schedule was 24 h of light during the first week, 18 h of light and 6 h of dark at the age of 3–14 days, followed by a gradual increase in the number of hours of light, reaching 20 h of light and 4 h of dark at the age of 21 days, which was maintained until they reached slaughter age.

For a contaminated diet, mycotoxin levels in broiler diets were obtained by spraying a liquid medium of mixed mycotoxin solutions on the feed [10]. The product was contaminated with highly concentrated AFB1, T-2, OTA, ZEA, and DON in approximately 100 µg kg^−1^ feed. However, the levels of mycotoxin contamination are shown in Table 10. The contaminated feed was left overnight at room temperature for the solvent to evaporate. The AB1, T-2, OTA, ZEA, and DON were obtained from the company R-Biopharm, Darmstadt, Germany (Trilogy Dried Standard No. TS-104, TS-314, TS-503, TS-401, and TS-310, respectively, USA). 

### 5.4. Growth Performance

Body weight gain (BWG) and feed consumption were determined from the broilers each pen at the start and end of each period. The average daily gain (ADG) and feed–conversion ratio (FCR) were calculated. Mortality was recorded daily, and the obtained data were calculated for the percentage of viability. Moreover, the amount of feed that was consumed per replicate was reported daily. The uneaten feed was thrown away, and new feed was given to the animals. The average daily feed intake (ADFI) was determined.

ADG (g) = BWG/(days of growth period × number of broilers)
ADFI (g) = feed consumption/(days of growth period × number of broilers)
FCR = feed consumption (kg)/total weight gain (kg)

### 5.5. Blood Collection and Analysis

Blood samples were collected from the wing veins of six chicks per treatment at the end of the experiment. The collected blood samples were then centrifuged at 2000× *g* for 15 min at room temperature, and the serums were gathered. The serums were stored (−20 °C) until their use. Serum samples were analyzed for kidney function tests (blood urea nitrogen (BUN), total protein (TP), albumin, and creatinine), liver function tests (aminotransferase (ALT)), and alkaline phosphatase (ALP) using a previous method by Mohaghegh et al. [38]. Moreover, the antioxidant levels of the serum (superoxide dismutase; SOD) were measured using the protocol in [68]. 

Blood samples were used for determining the mycotoxin residues. One hundred and fifty µL of chicken plasma was added in 96-well plate, as suggested by Lauwers et al. [47]. Next, 15 µL of a 100 ng mL^−1^ working solution (WSmix) were added, the mixture and equilibration was pipetted slowly up and down for five minutes at room temperature. Next, 450 µL of Acetonitrile with 0.1% (*v*/*v*) formic acid were added. The well plate had been gently mixed, and it was put under vacuum to enable the sample to pass through it. Each sample’s eluate was transferred into a polypropylene tube, dried under a nitrogen stream at 40 ± 5 °C, and reconstituted in 150 µL of MeOH/water (85/15; *v*/*v*). An aliquot of 5 µL was injected onto the analytical instruments.

### 5.6. Mycotoxin Analysis

Mycotoxins in blood samples were analysed using LC-MS/MS system (6545 Q-TOF, Agilent Technology, Wilmington, DE, USA) by following the reported method by Brezina et al. [46]. The following parameters were evaluated: linearity, within-day and between-day precision and accuracy, limit of quantification (LOQ), carry over, specificity, extraction recovery, and matrix effects. Recovery experiments were carried out with serum samples of broiler chickens that were spiked at three concentration ranges of 1–200 ng mL^−1^ in six replicates (*n* = 72) in the case of the serum. The LOD and LOQ for each analyte were calculated based on the signal-to-noise ratios (S/N), which were ≥3 and ≥10, respectively. The recoveries and the limits of detection (LOD) values of the mycotoxin tests for AFB1, ZEN, OTA, T-2, and DON were 0.005, 0.005, 0.009, 0.002, and 0.006 ng mL^−1^, respectively, and limits of quantification (LOQ) values of each mycotoxin test were 0.016, 0.014, 0.029, 0.007, and 0.018 ng mL^−1^, respectively.

### 5.7. The Relative Organ Weights

At the end of the experiment, one bird was randomly selected from each replicate (6 birds per treated group) for carcass and visceral organ evaluation. The weight of the internal organs (spleens, kidneys, livers, hearts, and pancreases) as percentages of the slaughter weight and relative organ weight was calculated. Finally, the relative organ weights were computed (g of organ 100 g^−1^ of live body weight) [45].

### 5.8. Immunohistochemistry of Liver Tissue

The representative samples of livers were collected in 10% neutral buffered formalin for histopathological examination. The tissues were fixed and processed by a routine paraffin embedding technique. To determine the presence of apoptosis in situ, section of 3–4 μm were taken. Apoptosis in liver cells was determined through the TUNEL technique, using the Apoptag plus peroxidase in situ kit (cod. S7101; Millipore Corporation, Billerica, MA, USA), according to the manufacturer’s instructions. The material was incubated with K proteinase for 30 min at room temperature and after that, endogenous peroxidase was blocked with H_2_O_2_ for 5 min. The reagents in this kit are designated to mark the free DNA 3’ OH terminal in situ with marked nucleotides. Nucleotides that are contained in the buffer reaction are enzymately connected to DNA by deoxynucleotidil transferase (TdT). Incubation with TdT was conducted at 37 °C for 60 min and the enzyme catalyzed an addition of triphosphate nucleotides to the final 3′ OH of the double or single helix DNA. Thereafter, incorporated nucleotides formed an oligomer randomly by digoxigenin. After that, rinsing with buffer solution was conducted for 30 min, and then, the anti-digoxigenin antibody was applied and incubated in a damp environment for 30 min at room temperature. Detection of the antigen-antibody link was made through the use of immunoperoxidase followed by DAB cromogen. Castrated mice prostates were used as a positive control. The omission of TdT enzyme during the TUNEL technique was used as a negative control, and this resulted in the non-coloring of the plate [69]. The histological sections were evaluated (40× magnification) using a compound microscope (A1 Zeiss Axio Scope, Oberkochen, Germany) and visualized using a high-quality digital camera (Canon EOS-6D mark, Canon USA, Inc., Huntington, New York, USA). Foci containing apoptotic nuclei were counted in the *in situ* tailing (IST)-stained sections of the livers. For counting the positive IST staining, 10 fields were used as random microscopic fields (40X), selecting the central tumor regions and avoiding necrosis areas, using the Sigma Scan Pro 5 imaging program. For calculating all of the indexes, the following formula was used: number of stained cells/1000 total cells.

### 5.9. Cecal Microbial Population

Fresh cecum digesta samples were taken for bacterial analyses within an hour according to the previous study by Giannenas et al. [70]. Briefly, *Lactobacillus* sp., *Bifidobacterium* sp. and *E. coli* in digesta samples were counted using traditional microbiological techniques and selective agar media after being serially diluted in 0.85 percent sterile saline solution. All of the microbiological tests were carried out in duplicate, with the average results being used for statistical analysis.

### 5.10. Intestinal Morphology

The samples of small intestine were extracted after euthanasia. They were then divided into duodenal, jejunal, and ileal segments. The small intestine was sectioned and cut into 2.0–3.0 cm for each segment, then washed with ice-cold phosphate-buffered saline. The segments were fixed in the 10% NBF (neutral buffered formalin) for 24 h. After fixation, the small intestinal tissue was dehydrated in increasing concentrations of ethanol solution (from 10% to absolute (100%)), then infiltrated with xylene, and embedded in paraffin wax. The microtome machine (Medite model A550, Medical GmbH, Burgdorf, Niedersachsen, Germany) was used to cut each segment into a thickness of 4 µm according to Srinual et al. [71]. They were then stained with H and E. Afterward, 60 images per treatment were examined for a study of the intestinal morphology. The histological sections were detected (10 × magnification) using a compound microscope (A1 Zeiss Axio Scope, Carl Zeiss, Gottingen, Germany). After that, Motic Images Plus 2.0 software (Motic China Group Co., Xiamen, Fujian, China) was used for a quantified morphometric analysis (villus height, villus width, crypt depth, muscularis mucosae thickness, villus height per crypt depth ratio, and villus surface area).

### 5.11. Statistical Analysis

All of the data were analyzed using the ANOVA with a 3 × 4 factorial designs using GLM procedures using SPSS 23.0 (SPSS Inc., Chicago, IL, USA). The main effect (levels of mycotoxin and fungal toxin adsorption) and interaction between the two factors were carried out. Duncan’s test was applied when any of the interactions showed significance. The pen was the experimental unit. Data are shown as the pooled mean ± SEM. The results were considered significantly different at *p* ≤ 0.05 and 0.0001.

## Figures and Tables

**Table 1:** Effect of mycotoxins and binders on the growth performance of broilers.

Toxins Diets	Adsorbent Level (g kg^−1^)	Growth Performance					
		Initial Weight (g)	Final Weight (g)	ADG ^1^ (g)	ADFI (g)	FCR	Mortality (%)
**8 to 21 d of age**							
CON ^2^	0	184.42	550.78	26.17	38.95 ^a^	1.49	2.50
	CB 0.5	184.29	568.29	27.43	38.32 ^a^	1.40	0.00
	RY 0.5	182.96	584.54	28.68	39.11 ^a^	1.36	0.00
	RY 1.0	182.58	601.71	29.94	38.50 ^a^	1.29	0.00
50% MT	0	183.63	530.83	24.80	37.96 ^ab^	1.53	5.00
	CB 0.5	182.92	535.00	25.14	38.12 ^a^	1.52	0.00
	RY 0.5	184.29	542.58	25.59	38.38 ^a^	1.50	0.83
	RY 1.0	184.79	555.04	26.45	37.89 ^ab^	1.43	0.00
100% MT	0	184.42	517.19	23.77	36.41 ^b^	1.53	7.50
	CB 0.5	183.67	557.50	26.70	38.01 ^ab^	1.42	0.00
	RY 0.5	184.08	557.29	26.66	38.13 ^a^	1.43	0.00
	RY 1.0	184.63	561.16	26.90	37.94 ^ab^	1.41	0.00
SEM ^3^		0.74	4.06	0.28	0.11	0.05	0.33
Source of variation		*p*-value					
MT		0.945	<0.001	<0.001	<0.001	0.210	0.288
Adsorbent		0.995	0.003	0.002	0.067	0.051	<0.001
MT × Adsorbent		0.995	0.783	0.703	0.040	0.890	0.282
**21 to 35 d of age**							
CON ^2^	0	550.78	1335.02	56.02	66.23	1.18	0.00 ^c^
	CB 0.5	568.29	1377.76	57.82	68.59	1.19	0.83 ^c^
	RY 0.5	584.54	1409.25	58.91	67.62	1.15	0.00 ^c^
	RY 1.0	601.71	1460.74	61.36	69.43	1.13	1.67 ^c^
50% MT	0	530.83	1255.96	51.79	67.81	1.31	9.47 ^b^
	CB 0.5	535.00	1331.36	56.88	67.79	1.19	2.50 ^c^
	RY 0.5	542.58	1360.53	58.42	66.40	1.14	0.83 ^c^
	RY 1.0	555.04	1390.24	59.66	67.53	1.13	0.83 ^c^
100% MT	0	517.19	1138.28	44.36	65.71	1.48	14.24 ^a^
	CB 0.5	557.50	1330.09	55.19	68.84	1.25	0.83 ^c^
	RY 0.5	557.29	1343.07	56.13	66.92	1.19	0.83 ^c^
	RY 1.0	561.16	1364.66	57.39	68.61	1.20	1.67 ^c^
SEM ^3^		4.06	9.64	0.53	0.31	0.02	0.41
Source of variation		*p*-value					
MT		<0.001	<0.001	<0.001	0.723	0.096	0.001
Adsorbent		0.003	<0.001	<0.001	0.061	0.461	<0.001
MT × Adsorbent		0.783	0.089	0.221	0.655	0.979	<0.001
**8 to 35 d of age**							
CON ^2^	0	184.42	1335.02 ^b^	41.09 ^b^	52.59	1.28	2.50 ^c^
	CB 0.5	184.29	1377.76 ^b^	42.62 ^ab^	53.45	1.25	0.83 ^c^
	RY 0.5	182.96	1409.25 ^ab^	43.79 ^ab^	53.37	1.22	0.00 ^c^
	RY 1.0	182.58	1460.74 ^a^	45.65 ^a^	53.96	1.18	1.67 ^c^
50% MT	0	183.63	1255.96 ^c^	38.30 ^c^	52.88	1.38	14.47 ^b^
	CB 0.5	182.92	1331.36 ^bc^	41.02 ^b^	52.99	1.29	2.50 ^c^
	RY 0.5	184.29	1360.53 ^b^	42.01 ^b^	52.39	1.25	1.67 ^c^
	RY 1.0	184.79	1390.24 ^ab^	43.05 ^ab^	52.71	1.22	0.83 ^c^
100% MT	0	184.42	1138.28 ^d^	34.07 ^d^	51.06	1.50	21.74 ^a^
	CB 0.5	183.67	1330.09 ^bc^	40.94 ^b^	53.42	1.30	0.83 ^c^
	RY 0.5	184.08	1343.07 ^b^	41.39 ^b^	52.52	1.27	0.83 ^c^
	RY 1.0	184.63	1364.66 ^b^	42.14 ^b^	53.28	1.26	1.67 ^c^
SEM ^3^		0.74	9.64	0.26	0.16	0.02	0.49
Source of variation		*p*-value					
MT		0.945	<0.001	<0.001	0.120	0.066	<0.001
Adsorbent		0.995	<0.001	<0.001	0.041	0.008	<0.001
MT × Adsorbent		1.000	<0.001	<0.001	0.063	0.052	<0.001

^a–d^ Means without the same superscripts in the same column differ (*p* ≤ 0.05). ^1^ ADG: average daily weight gain; ADFI: average daily feed intake; FCR: feed conversion ratio; ^2^ CON: control diet; MT: mycotoxin; CB: commercial mycotoxin binder; RY: red yeast. ^3^ SEM: standard error of the mean.

**Table 2:** Effect of mycotoxins and binders on the blood chemistry of broilers.

Toxins Diets	Adsorbent level (g kg^−1^)		Blood Chemistry					
		ALT ^1^	Albumin	ALP	CL	TP	BUN	SOD
CON ^2^	0	1.67	0.97	1364.17	0.26	2.60	1.93	0.33
	CB 0.5	1.67	0.87	1412.83	0.25	2.60	1.82	0.42
	RY 0.5	1.00	0.97	1521.33	0.27	2.80	2.30	0.50
	RY 1.0	0.67	0.93	1572.17	0.27	2.58	1.80	0.51
50% MT	0	1.83	0.83	1789.83	0.26	2.43	1.95	0.10
	CB 0.5	0.83	0.97	1612.00	0.25	2.90	2.22	0.33
	RY 0.5	0.17	0.98	1607.67	0.26	2.87	2.03	0.47
	RY 1.0	0.67	0.93	1639.33	0.25	2.65	1.92	0.19
100% MT	0	2.17	1.02	1988.00	0.28	2.90	2.03	0.26
	CB 0.5	0.67	1.03	1559.83	0.27	2.92	1.78	0.22
	RY 0.5	0.67	0.93	1504.67	0.26	2.62	1.92	0.47
	RY 1.0	0.50	1.05	1445.50	0.26	2.90	2.03	0.37
SEM ^3^		0.08	0.02	76.71	0.01	0.04	0.04	0.02
Source of variation		*p*-value						
MT		<0.001	0.118	0.550	0.113	0.158	0.650	0.199
Adsorbent		0.451	0.922	0.811	0.596	0.523	0.471	0.202
MT × Adsorbent		0.670	0.303	0.883	0.365	0.139	0.111	0.106

^1^ ALT: alanine aminotransferase; ALP: alkaline phosphatase; CL: creatinine; TP: total protein; BUN: blood urea nitrogen; SOD: superoxide dismutase; ^2^ CON: control diet; MT: mycotoxin; CB: commercial mycotoxin binder; RY: red yeast. ^3^ SEM: standard error of the mean. *n* = six chicks/group.

**Table 3:** The concentrations of mycotoxins in blood samples of broilers.

Toxins F8Diets	Adsorbent Level (g kg^−1^)	Mycotoxin in Blood Samples				
		AFB1 ^3^	ZEN	OTA	T-2	DON
CON ^1^	0	ND	ND	ND	ND	ND
	CB 0.5	ND	ND	ND	ND	ND
	RY 0.5	ND	ND	ND	ND	ND
	RY 1.0	ND	ND	ND	ND	ND
50% MT	0	0.45 ^b^	1.13 ^b^	1.84 ^b^	0.62 ^b^	0.84 ^b^
	CB 0.5	0.05 ^d^	0.01 ^c^	0.01 ^d^	0.01 ^c^	0.13 ^c^
	RY 0.5	0.12 ^c^	0.04 ^c^	0.05 ^d^	0.01 ^c^	0.03 ^d^
	RY 1.0	0.13 ^c^	0.01 ^c^	0.01 ^d^	0.01 ^c^	0.12 ^c^
100% MT	0	1.26 ^a^	2.74 ^a^	4.32 ^a^	1.04 ^a^	1.08 ^a^
	CB 0.5	0.32 ^a^	0.04 ^c^	0.08 ^d^	0.01 ^c^	0.12 ^c^
	RY 0.5	0.13 ^c^	0.01 ^c^	0.47 ^c^	0.01 ^c^	0.07 ^d^
	RY 1.0	0.10 ^c^	0.04 ^c^	0.18 ^d^	0.03 ^c^	0.08 ^cd^
SEM ^2^		0.01	0.02	0.02	0.01	0.01
Source of variation		*p*-value				
MT		<0.001	<0.001	<0.001	<0.001	<0.001
Adsorbent		<0.001	<0.001	<0.001	<0.001	<0.001
MT × Adsorbent		<0.001	<0.001	<0.001	<0.001	<0.001

^a–d^ Means without the same superscripts in the same column differ (*p* ≤ 0.05). ^1^ CON: control diet; MT: mycotoxin; CB: commercial mycotoxin binder; RY: red yeast. ^2^ SEM: standard error of the mean. ^3^ AFB1: Aflatoxin B1; ZEN: Zearalenone; OTA: Ochratoxin A; T-2: T-2 toxin; DON: Deoxynivalenol; ND: not detected. *n* = six chicks/group.

**Table 4:** Effect of mycotoxins and binders on the pathology characteristics and relative organ weight of broilers.

Toxins Diets	Adsorbent Level (g kg^−1^)	Relative Organ Weight (%)				
		Spleen	Kidney	Liver	Heart	Pancreases
CON ^1^	0	0.13	0.44 ^c^	2.33 ^b^	0.71	0.27 ^c^
	CB 0.5	0.16	0.70 ^ab^	2.63 ^b^	0.80	0.36 ^bc^
	RY 0.5	0.14	0.71 ^ab^	2.49 ^b^	0.77	0.33 ^c^
	RY 1.0	0.16	0.67 ^ab^	2.53 ^b^	0.73	0.36 ^bc^
50% MT	0	0.15	0.74 ^ab^	3.01 ^b^	0.67	0.44 ^b^
	CB 0.5	0.15	0.72 ^ab^	2.83 ^b^	0.77	0.33^c^
	RY 0.5	0.15	0.62 ^b^	2.64 ^b^	0.69	0.32 ^c^
	RY 1.0	0.12	0.68 ^ab^	2.58 ^b^	0.81	0.34 ^c^
100% MT	0	0.12	0.81 ^a^	3.96 ^a^	0.60	0.53 ^a^
	CB 0.5	0.12	0.57 ^bc^	2.99 ^b^	0.79	0.31 ^c^
	RY 0.5	0.11	0.59 ^bc^	2.67 ^b^	0.66	0.31 ^c^
	RY 1.0	0.15	0.60 ^bc^	2.50 ^b^	0.74	0.33 ^c^
SEM ^2^		0.01	0.01	0.07	0.01	0.01
Source of variation		*p*-value				
MT		0.240	0.265	0.011	0.191	0.106
Adsorbent		0.806	0.950	0.027	0.003	0.001
MT × Adsorbent		0.287	<0.001	0.038	0.434	<0.001

^a–c^ Means without the same superscripts in the same column differ (*p* ≤ 0.05). ^1^ CON: control diet; MT: mycotoxin; CB: commercial mycotoxin binder; RY: red yeast. ^2^ SEM: standard error of the mean. *n* = six chicks/group.

**Table 5:** Effect of mycotoxins and binders on the cecum bacteria populations of broilers.

Toxins Diets	Adsorbent Level (g kg^−1^)	Bacteria Populations (log^10^ CFU g^−1^)		
		*E. coli*	*Lactobacillus* sp.	*Bifidobacterium* sp.
CON ^1^	0	5.98 ^e^	6.47 ^b^	5.81 ^b^
	CB 0.5	5.81 ^e^	7.24 ^a^	5.50 ^c^
	RY 0.5	7.12 ^b^	7.13 ^a^	6.04 ^ab^
	RY 1.0	6.23 ^d^	7.58 ^a^	6.28 ^a^
50% MT	0	7.04 ^b^	5.82 ^d^	4.98 ^d^
	CB 0.5	6.72 ^c^	6.12 ^c^	5.47 ^c^
	RY 0.5	5.96 ^e^	6.38 ^b^	5.78 ^b^
	RY 1.0	5.26 ^f^	6.56 ^b^	6.16 ^a^
100% MT	0	7.69 ^a^	4.24 ^g^	4.08 ^e^
	CB 0.5	7.00 ^b^	5.24 ^ef^	5.08 ^d^
	RY 0.5	7.08 ^b^	5.17 ^f^	5.11 ^d^
	RY 1.0	6.41 ^d^	5.43 ^e^	5.14 ^d^
SEM ^2^		0.02	0.02	0.02
Source of variation		*p*-value		
MT		<0.001	<0.001	<0.001
Adsorbent		<0.001	<0.001	<0.001
MT × Adsorbent		<0.001	0.004	<0.001

^a–g^ Means without the same superscripts in the same column differ (*p* ≤ 0.05). ^1^ CON: control diet; MT: mycotoxin; CB: commercial mycotoxin binder; RY: red yeast. ^2^ SEM: standard error of the mean. *n* = six chicks/group.

**Table 6:** Effect of mycotoxins and binders on the duodenum morphology of broilers.

Toxins Diets	Adsorbent Level (g kg^−1^)	Duodenum Morphology					
		VH ^1^(µm)	VW (µm)	CD (µm)	MMT (µm)	VH:CD	VSA (mm^2^)
CON ^2^	0	4681.71 ^e^	464.22 ^d^	603.52 ^bc^	84.09 ^cd^	7.85 ^cd^	6.87 ^f^
	CB 0.5	5239.65 ^d^	521.72 ^cd^	556.56 ^bc^	65.74 ^ef^	9.77 ^abc^	8.64 ^de^
	RY 0.5	5727.52 ^bc^	488.72 ^cd^	677.41 ^abc^	64.00 ^ef^	9.13 ^bc^	8.78 ^de^
	RY 1.0	6109.09 ^a^	547.70 ^bc^	535.46 ^c^	62.06 ^ef^	11.76 ^a^	10.51 ^ab^
50% MT	0	5497.58 ^d^	476.29 ^d^	667.92 ^abc^	120.64 ^a^	8.34 ^cd^	8.22 ^de^
	CB 0.5	5735.48 ^bc^	623.02 ^a^	614.96 ^abc^	105.95 ^b^	10.85 ^ab^	11.23 ^a^
	RY 0.5	5646.32 ^bc^	638.80 ^a^	711.66 ^ab^	71.88 ^de^	8.21 ^cd^	11.35 ^a^
	RY 1.0	5892.72 ^ab^	594.72 ^ab^	601.35 ^bc^	56.28 ^ef^	10.59 ^ab^	11.01 ^a^
100% MT	0	5179.44 ^d^	497.21 ^cd^	774.36 ^a^	101.72 ^b^	6.70 ^d^	8.08 ^e^
	CB 0.5	5742.89 ^bc^	508.08 ^cd^	574.42 ^bc^	91.56 ^bc^	10.63 ^ab^	9.15 ^cde^
	RY 0.5	5938.88 ^ab^	549.19 ^bc^	559.76 ^bc^	51.92 ^f^	10.82 ^ab^	10.28 ^abc^
	RY 1.0	5885.79 ^ab^	511.34 ^cd^	642.44 ^abc^	65.77 ^ef^	9.39 ^bc^	9.44 ^bcd^
SEM ^3^		33.73	5.66	10.07	1.49	0.18	0.12
Source of variation		*p*-value					
MT		0.003	<0.001	0.060	<0.001	0.867	<0.001
Adsorbent		<0.001	<0.001	0.001	<0.001	<0.001	<0.001
MT × Adsorbent		<0.001	0.001	0.001	<0.001	0.004	0.010

^a–f^ Means without the same superscripts in the same column differ (*p* ≤ 0.05). ^1^ VH: villus height; VW: villus width; CD: crypt depth; MMT: muscularis mucosae thickness; VH:CD: villus height per crypt depth ratio; VSA: villus surface area. ^2^ CON: control diet; MT: mycotoxin; CB: commercial mycotoxin binder; RY: red yeast. ^3^ Pooled SEM: standard error of the mean. *n* = six chicks/group.

**Table 7:** Effect of mycotoxins and binders on the jejunum morphology of broilers.

Toxins Diets	Adsorbent Level (g kg^−1^)	Jejunum Morphology					
		VH ^1^ (µm)	VW (µm)	CD (µm)	MMT (µm)	VH:CD	VSA (mm^2^)
CON ^2^	0	4756.42 ^d^	511.46	551.77 ^c^	80.95 ^bc^	9.68 ^a^	7.65
	CB 0.5	5772.65 ^a^	420.46	669.32 ^ab^	79.52 ^bc^	8.95 ^a^	7.64
	RY 0.5	4502.82 ^de^	447.13	551.03 ^c^	89.41 ^bc^	8.63 ^a^	6.46
	RY 1.0	5599.24 ^a^	500.23	616.07 ^bc^	80.98 ^bc^	9.56 ^a^	8.74
50% MT	0	4148.24 ^e^	471.166	526.90 ^c^	99.81 ^b^	7.89 ^ab^	6.14
	CB 0.5	4807.77 ^cd^	447.21	537.97 ^c^	71.87 ^c^	9.18 ^a^	6.80
	RY 0.5	4519.19 ^de^	402.66	563.37 ^c^	89.84 ^bc^	8.40 ^a^	5.70
	RY 1.0	5381.33 ^ab^	432.62	594.32 ^bc^	94.52 ^bc^	9.72 ^a^	7.32
100% MT	0	4296.48 ^e^	462.96	724.50 ^a^	130.66 ^a^	6.22 ^b^	6.26
	CB 0.5	5203.37 ^c^	455.07	625.78 ^bc^	83.10 ^bc^	8.49 ^a^	7.55
	RY 0.5	5480.09 ^b^	419.39	609.82 ^bc^	102.84 ^b^	9.11 ^a^	7.19
	RY 1.0	5476.95 ^ab^	440.97	572.36 ^bc^	94.12 ^bc^	9.88 ^a^	7.60
SEM ^3^		41.57	5.02	9.28	1.56	0.17	0.11
Source of variation		*p*-value					
MT		<0.001	0.028	0.004	<0.001	0.195	<0.001
Adsorbent		<0.001	<0.001	0.564	0.323	0.005	<0.001
MT × Adsorbent		<0.001	0.077	0.002	<0.001	0.029	0.103

^a–e^ Means without the same superscripts in the same column differ (*p* ≤ 0.05). ^1^ VH: villus height; VW: villus width; CD: crypt depth; MMT: muscularis mucosae thickness; VH:CD: villus height per crypt depth ratio; VSA: villus surface area; ^2^ CON: control diet; MT: mycotoxin; CB: commercial mycotoxin binder; RY: red yeast. ^3^ SEM: standard error of the mean. *n* = six chicks/group.

**Table 8:** Effect of mycotoxins and binders on the ileum morphology of broilers.

Toxins Diets	Adsorbent Level (g kg^−1^)	Ileum Morphology					
		VH ^1^ (µm)	VW (µm)	CD (µm)	MMT (µm)	VH:CD	VSA (mm^2^)
CON ^2^	0	2753.69 ^f^	468.36 ^a^	436.71 ^b^	112.02 ^cd^	6.51 ^bc^	4.01
	CB 0.5	3663.17 ^ab^	394.18 ^cd^	533.53 ^ab^	134.44 ^b^	7.05 ^ab^	4.57
	RY 0.5	3950.62 ^a^	440.426 ^ab^	505.99 ^b^	113.33 ^bcd^	7.91 ^a^	5.53
	RY 1.0	3548.76 ^abc^	422.34 ^bc^	442.93 ^b^	115.83 ^bcd^	8.11 ^a^	4.71
50% MT	0	3042.28 ^ef^	318.40 ^ef^	527.51 ^ab^	109.74 ^cd^	6.03 ^bc^	3.05
	CB 0.5	2804.32 ^ef^	364.01 ^de^	497.33 ^b^	112.04 ^cd^	6.02 ^bc^	3.21
	RY 0.5	3365.45 ^bcd^	331.80 ^ef^	520.66 ^ab^	118.93 ^bc^	6.75 ^ab^	3.52
	RY 1.0	3192.44 ^cde^	355.04 ^de^	488.37 ^b^	113.66 ^bcd^	6.88 ^ab^	3.62
100% MT	0	2940.70 ^ef^	302.63 ^f^	611.42 ^a^	178.54 ^a^	5.33 ^c^	2.79
	CB 0.5	3286.89 ^bcd^	361.03 ^de^	432.99 ^b^	105.09 ^cd^	7.89 ^a^	3.69
	RY 0.5	3502.98 ^bc^	335.90 ^ef^	485.68 ^b^	95.46 ^de^	7.90 ^a^	3.70
	RY 1.0	3478.54 ^bc^	325.36 ^ef^	527.62 ^ab^	84.72 ^e^	6.99 ^ab^	3.54
SEM ^3^		26.31	4.15	9.02	1.98	0.12	0.06
Source of variation		*p*-value					
MT		<0.001	<0.001	0.248	0.550	0.005	<0.001
Adsorbent		<0.001	0.863	0.392	<0.001	<0.001	<0.001
MT × Adsorbent		<0.001	<0.001	0.002	<0.001	0.048	0.073

^a–f^ Means without the same superscripts in the same column differ (*p* ≤ 0.05). ^1^ VH: villus height; VW: villus width; CD: crypt depth; MMT: muscularis mucosae thickness; VH:CD: villus height per crypt depth ratio; VSA: villus surface area; ^2^ CON: control diet; MT: mycotoxin; CB: commercial mycotoxin binder; RY: red yeast. ^3^ SEM: standard error of the mean. *n* = six chicks/group.

**Table 9:** Composition of basal diet.

Ingredient (g kg^−1^ Unless Stated Otherwise)	Starter (1 to 21 Days)	Grower (22 to 35 Days)
Ground yellow corn (7.8% CP)	47.70	46.00
Soybean meal (44% CP)	26.12	24.95
Full-fat soy bean (36% CP)	18.00	20.00
Dicalcium phosphate	1.70	1.30
Salt (NaCl)	0.30	0.35
Vitamin Premix ^1^	0.05	0.05
Mineral Premix ^2^	0.10	0.10
Corn oil	4.40	6.00
Limestone	1.10	0.80
Choline	0.05	0.05
L-Lysine	0.32	0.25
DL-methionine	0.16	0.15
Total	100.00	100.00
**Calculated chemical analysis**		
Crude protein (%)	22.52	20.07
Metabolisable energy (kcal kg^−1^)	3121	3175
Calcium (%)	1.12	1.13
Available phosphorus (%)	0.52	0.53
Lysine (%)	1.42	1.30
Methionine (%)	0.67	0.56
Methionine and cystine (%)	1.06	0.91
Tryptophan (%)	0.29	0.25
Threonine (%)	0.90	0.80

^1^ Vitamin premix (per kg premix): vitamin A 20,000,000 IU, vitamin D3 4,000,000 IU, vitamin E 11,000 IU, vitamin K3 4.00 g, vitamin B1 5.00 g, vitamin B2 10.00 g, vitamin B6 5.00 g, vitamin B12 0.02 g, vitamin C 15.00 g, pantothenic acid 15.00 g, folic acid 3.00 g, nicotinic acid 40.00 g, biotin 0.20 g. ^2^ Mineral premix (per kg premix): magnesium 100.00 g, potassium 90.00 g, sodium 100.00 g, and feed additive 25.30 g.

**Table 10:** The levels of mycotoxins in experimental diet.

Mycotoxin Name	Assigned Value							Coefficient
	50 µg kg^−1^ (50% MT)			100 µg kg^−1^ (100% MT)				
	Mean (µg kg^−1^) ± SD	RSD (%)	CV	Mean (µg kg^−1^) ± SD		RSD (%)	CV	
AFB1	49.86 ± 0.09	0.18	0.000	99.08	± 0.09	0.09	0.000	0.988
ZEN	49.95 ± 2.63	5.27	0.005	99.66	± 0.85	0.85	0.001	0.989
OTA	49.93 ± 0.29	0.58	0.001	99.98	± 0.00	0.00	0.000	0.985
T-2	49.88 ± 4.72	9.46	0.009	99.97	± 0.18	0.18	0.000	0.990
DON	49.96 ± 1.05	2.10	0.002	99.03	± 0.50	0.50	0.001	0.982

## Data Availability

Data are available on request due to restrictions, e.g., privacy or ethical restrictions. The data presented in this study are available on request from the corresponding author. The data are not publicly available due to the law of the Ministry of Higher Education, Science, Research and Innovation.
